# On inflammatory hypothesis of depression: what is the role of IL-6 in the middle of the chaos?

**DOI:** 10.1186/s12974-021-02100-7

**Published:** 2021-02-16

**Authors:** Elnaz Roohi, Nematollah Jaafari, Farshad Hashemian

**Affiliations:** 1 Department of Clinical Pharmacy, Faculty of Pharmacy, Tehran Medical Sciences, Islamic Azad University, No. 99 Yakhchal Street, Shariati Avenue, Tehran, 1941933111 Iran; 2 Université de Poitiers, Unité de recherche clinique intersectorielle Pierre Deniker du Centre Hospitalier Henri Laborit F-86022 France, Groupement De Recherche CNRS 3557, Poitiers, France

**Keywords:** Neuroinflammation, Major depressive disorder, Interleukin-6, Cytokines

## Abstract

Many patients with major depressive disorder (MDD) are reported to have higher levels of multiple inflammatory cytokines including interleukin 6 (IL-6). Recent studies both pre-clinical and clinical have advocated for the functional role of IL-6 in development of MDD and suggested a great potential for targeting this cytokine to open new avenues in pharmacotherapy of depression. The purpose of the present narrative review was to provide an integrated account of how IL-6 may contribute to development of depression. All peer-reviewed journal articles published before July 2020 for each area discussed were searched by WOS, PubMed, MEDLINE, Scopus, Google Scholar, for original research, review articles, and book chapters. Publications between 1980 and July 2020 were included. Alterations in IL-6 levels, both within the periphery and the brain, most probably contribute to depression symptomatology in numerous ways. As IL-6 acts on multiple differing target tissues throughout the body, dysregulation of this particular cytokine can precipitate a multitude of events relevant to depression and blocking its effects can prevent further escalation of inflammatory responses, and potentially pave the way for opening new avenues in diagnosis, treatment, and prevention of this debilitating disorder.

## Background

Major depressive disorder (MDD) is a leading cause of disability throughout the world with a global prevalence of 2.6–5.9% [1]. The total estimated number of people living with depression worldwide increased by 49.86% from 1990 to 2017 [2]. According to worldwide projections, MDD will be the single major cause of burden of all health conditions by 2030 [3]. MDD is characterized by periods of low mood, altered cognition, considerable functional burden including impaired occupational functioning and psychosocial disability [4]. Despite available pharmacotherapeutic options, 30–60% of patients with MDD are not responsive to available treatments [5] and the rate of remission of the disease is often < 50% [6], while recurrence rates are more than 85% within 10 years of a depressive episode, and average about ≥ 50% within 6 months of assumed clinical remission [4]. Indeed, there exists no compelling evidence that current treatments are capable of disease modification in MDD patients. Thus, therapeutic deficiency in treatment outcomes reflects the demand for revitalizing psychiatric therapeutics with novel pharmacotherapeutic options that engage non-monoaminergic molecular targets.

A large body of evidence suggests that inflammation has central role in pathogenesis of MDD [7–13]. However, the exact mechanisms underlying inflammation-induced depression are not completely elucidated [3]. Historically, the “monoamine-depletion hypothesis” has been the main proposed pathophysiology [14]; nevertheless, this hypothesis alone cannot fully account for pathogenesis of MDD [15, 16]. In recent years, “inflammatory hypothesis” has been proposed [17]. However, it is noteworthy that it was probably in the early 1990s that for the first time, possible relationships between the peripheral immune system and major depression was studied [18]. Maes et al. (1992) established immune cell profile of patients with depression and advocated for the existence of a systemic immune activation during major depressive disorder [19]. Moreover, correlations between IL-6 activity, acute phase proteins, and hyperactivity of the hypothalamic-pituitary-adrenal (HPA) axis were suggested in severe depression [20].

Most proximally, inflammation is regulated by expression of immune response genes including interleukin (IL)-1B, tumor necrosis factor (TNF), and IL-6 which promote secretion of pro-inflammatory cytokines leading to systemic inflammation. Distally, inflammation is regulated in the brain where socio-environmental cues including possible threat are detected. This neuro-inflammatory link can activate the conserved transcriptional response to adversity (CTRA) before happening of a possible threat or bacterial infection. However, the negative aspect of central regulation of systemic inflammation is that it can give social and foreseen dangers (including those that have not yet occurred or may never actually happen) the ability to activate the CTRA in the absence of actual physical danger. Under normal conditions, CTRA-related inflammatory activity is downregulated by the HPA axis via the production of cortisol. Nevertheless, when prolonged actual or perceived social threat or physical danger is present, glucocorticoid resistance can develop which leads to excessive inflammation that heightens a person’s risk for development of several disorders including MDD, especially if activation of these pathways is prolonged [21]. As mentioned above, the current understanding of MDD encloses not only alterations in neurotransmitters, but also changes in immune and endocrine functioning as well as neural circuits [22]. This broadened framework has just started to inform a wide array of novel, personalized therapeutics that are showcasing great promise in a new holistic approach to MDD [23].

Cytokines are implicated in pathogenesis of MDD [24–30]. Risk factors of developing MDD include familial, developmental, psychological, and medical risk factors as well as molecular factors associated with genetics, epigenetics, gene expression, and also those related to the endocrine and the immune system [31, 32]. All these risk factors have been shown to be related with changes in cytokine production or signaling. In other words, cytokines are involved in almost every predisposing or precipitating risk factor associated with MDD [24]. Indeed, there is accumulating evidence in favor of involvement of pro-inflammatory cytokines in pathophysiology of depression [24, 29, 33–36]. Various studies reported higher levels of multiple inflammatory markers including IL-6 in patients with MDD [37–41]. Of all pro-inflammatory cytokines, changes in IL-6 serum levels have been reported as one of the most reproducible abnormalities in MDD [38].

The aim of the present narrative review is to elucidate the fundamentals, implications, challenges of cytokine research specifically IL-6 in major depressive disorder. This comprises of the following:

-) A Brief overview of cytokines

-) Cytokine categories according to immunological function.

-) IL-6 as a pleiotropic cytokine.

-) Brief overview of chemokines and their role in Depression.

-) Challenges of cytokine research in psychiatry.

-) IL-6 alterations in depression.

-) Effects of IL-6 on neurotransmitters’ synthesis, signaling, metabolism, and function.

-) Effects of IL-6 levels on brain morphology in depression.

-) Blockade of IL-6 and its receptor in the periphery as a potential therapeutic option in MDD.

-) Possible role of IL-6 together with gut microbiota in pathogenesis of depression.

-) Elevated levels of IL-6 in patients with COVID-19 infection.

## Methods

The present article is a narrative review. All peer-reviewed journal articles published before July 2020 for each area discussed were searched by WOS, PubMed, MEDLINE, Scopus, Google Scholar, for original research, review articles, and book chapters. We selected articles on the basis of being comprehensive, innovative, and informative for an in-depth understanding and a critical debate on the topic. Publications between 1973 and 2020 were included.

### A brief overview of cytokines

Cytokines are a broad category of released proteins that act as signaling molecules to regulate inflammation and cellular activities [24, 42]. They are produced by different immune cells (e.g., macrophages, lymphocytes, mast cells), parenchymal cells, endothelial and epithelial cells, fibroblasts, adipocytes, and stromal cells within the periphery [24, 43]. Additionally, they are produced by microglia, astrocytes, and neurons in the brain [44]. Cytokines from the periphery (peripherally produced cytokines) can exert influences on inflammatory processes in the brain [45, 46]. Indeed, they can enter blood-brain barrier (BBB) and affect the brain via humoral (accessing the brain through leaky secretions of the BBB such as choroid plexus), neural (through stimulation of primary afferent nerve fibers in the vagus nerve), and cellular (through stimulation of microglia by pre-inflammatory cytokines to produce monocyte chemottractant protein-1 and recruit monocytes to the brain) pathways [47]. Most cytokines function in their immediate microenvironment. Few of them are involved in paracrine signaling which indeed is fundamental to the control of an inflammatory response within a given tissue or organ and the activation of a coordinated immune response that involves multiple cell types [48]. Apart from navigating the immune system to defend the body from pathogens, cytokines have a modifying effect on neurotransmission [49].

It’s also noteworthy that the same cytokines can be produced by multiple cell types. For example, white blood cells, endothelium, fat cells, and other cells can produce TNF-α [50]. Additionally, one single cell can release different cytokines. For instance, T Helper type 2 (T_H_2) cells can produce IL-3, IL-4, IL-5, IL-6, and IL-13 [24]. Cytokines can have pleiotropic, redundant, synergistic, and antagonistic effects [51]. The phenomenon that a single cytokine can act on several different cell types is called pleiotropy [51]. For instance, IL-10 can activate T_H_2 cells and B cells, yet inhibit macrophages and T helper type 1 (T_H_1) cells. Thus, being immunostimulatory as well as being immunosuppressive [52]. Cytokines are redundant in their activity, i.e., similar functions can be exerted by different cytokines. For instance, interferon (IFN)-γ, IL-2, and TNF-α enhance cellular immunity and production of cytotoxic cell contacts [53]. Cytokines can also act synergistically, i.e., they can have combined effects when acting together. For instance, IL-3 and IL-4 amplify each other’s effects to induce growth, differentiation, and activation of mast cells in a synergistic manner [24]. Another phenomenon in cytokines signaling is antagonism. An example of cytokine antagonism is that cytokines of the IL-1 superfamily can antagonize IL-18 effects [54].

### Cytokine categories according to immunological function

Four categories of cytokines are usually referred to in psychoimmunological literature. (1) T_H_1 cytokines (IL-2, IL-12, IFN-γ) which induce cytotoxic cell contacts. (2) T_H_2 cytokines (IL-4, (IL-5, IL-13) which lead to production of antibodies. (3) Pro-inflammatory cytokines (IL-1, IL-6, IL-8, IL-17, IL-21, IL-22, IFN-α, TNF-α) which further the progress of inflammation. (4) Anti-inflammatory cytokines (IL-10, transforming growth factor-beta (TGF)-β which are influenced by regulatory T cells and impede inflammatory process from escalating [24]. However, these categories are not distinct and it must be considered that cytokines can exert various effects on different cells and therefore, they may have pro- and also anti-inflammatory properties. For instance, IFN-α which has been listed as a pro-inflammatory cytokine can also have anti-inflammatory properties [55].

### IL-6 as a pleiotropic cytokine

IL-6 was first identified as a differentiation factor for B cells which stimulates production of antibodies by activated B cells. Apart from regulation of acute inflammation, IL-6 is known to induce differentiation of B cells, and activation and population expansion of T cells [56]. Within the peripheral and central nervous system (CNS), IL-6 can act as a neuronal growth factor inducing neurite development and nerve regeneration [57]. IL-6 receptor (IL-6R) consists of the IL-6-binding chain which has two forms of transmembrane IL-6R and soluble IL-6R (sIL-6R) [58] and a gp130 signal-transducing chain [59]. Following binding to its receptor (IL-6R), IL-6 initiates to exert its multiple functions.

It is quite interesting that IL-6 exerts both pro- and anti-inflammatory properties [60, 61]. Indeed, its signaling is complex and can lead to both inflammatory and anti-inflammatory cascades depending upon the presence of either IL-6 receptor (IL-6R) or the membrane bound gp130 signal transducer and these are expressed at very different frequencies within specific cell type in the body [5]. Trans-signaling of IL-6, in which the soluble form of the IL-6 receptor (sIL-6R) is shed from the membrane bound receptors, is known to be pro-inflammatory [62]. The sIL-6R binds to IL-6 and is transported to any cell type on which gp130 is expressed [63]. While most soluble receptors (e.g., soluble receptor for TNFα) result in antagonistic action by competing for the ligand, the sIL-6R is agonistic and increases the types of cells through which IL-6 can signal. Additionally, IL-6 engages in classical signaling which is anti-inflammatory [63] and occurs through binding of IL-6 to the membrane bound cell surface receptor. Classical signaling of IL-6 solely occurs on some subsets of T cells, neutrophils and monocytes megakaryocytes, and hepatocytes [64]. In both classical and trans-signaling, the IL-6/IL-6R/gp130 complex uses two pathways to activate intracellular signaling namely the Janus kinase/signal transducer and activator of transcription (JAK/STAT) pathway and the mitogen-activated protein kinase (MAPK) pathway [5].

Indeed, IL-6 has been mostly regarded as having pro-inflammatory properties; however, it has many anti-inflammatory functions which are necessary for resolution of inflammation [65]. For instance, IL-6 inhibits activity of the transcription factor named nuclear factor kappa-light-chain-enhancer of activated B cells (NF-κB) and expression of the chemokine receptor on dendritic cells which is needed for recruiting these cells to lymphoid tissues; thus, involving in resolution of inflammation [66]. Research findings showed that IL-6 has a crucial role in regulation of T helper17 (Th17)/regulatory T (Treg) cells [67]. In the presence of TGF-β, IL-6 is a vital signal for differentiation of naive T cells into Th17 cells which in turn are implicated in induction of autoimmune diseases [68, 69], and result in local tissue injury in chronic inflammatory disorders [70]. On the contrary, IL-6 can strongly inhibit the TGF-β-induced differentiation of Treg cells which in turn results in inhibition of autoimmunity and protects against tissue damage [71]. Functional dichotomy of IL-6 indicates that it may be responsible for maintaining the balance between pro- and anti-inflammatory responses, while having tissue-specific properties at the periphery and in the CNS [72].

### Brief overview of chemokines and their role in depression

Chemokines are small chemotactic cytokines that are identified to have significant roles in migration of immune cells, induction of direct chemotaxis, and propagation of inflammatory responses [73]. They are classified into four sub-families based on their structural criteria (i.e., the number and spacing of their two N-terminals, disulfide bonding participating cysteine residues). These four subfamilies include CXC, CC, C, and CX3C [74]. Furthermore, they can be categorized according to their biological activity, namely, the maintenance of homeostasis and the induction of inflammation. There are also chemokines which have dual functionality [75].

These small chemotactic cytokines are known to be secreted in response to inflammatory cytokines; thereafter, selectively recruiting lymphocytes, monocytes, and neutrophil-inducing chemotaxis by activating G-protein-coupled receptors (GPCRs) [76]. A growing body of evidence suggests that chemotactic cytokines are implicated in neurobiological processes relevant to psychiatric disorders such as synaptic transmission and plasticity, neuroglia communication, and neurogenesis [77]. Indeed, disruption of any of the mentioned functions which may take place by activation of the inflammatory response system has consistently been found to be relevant in pathogenesis of depression [73].

There are indeed both pre-clinical and clinical evidence in support of linking alterations in the chemokine network to depressive behavior [73]. In an animal model of depression, namely prenatal stressed rats, levels of CCL2, and CXCL12 chemokines were found to be upregulated in the hippocampus and prefrontal cortex, which was indeed suggestive of excessive microglial activation [78]. Additionally, Trojan et al. (2017) investigated the modulatory properties of chronic administration of anti-depressants on the chemokines. According to their results, chronic administration of anti-depressants has been shown to normalize the prenatal stress-induced behavioral disturbances together with the observed alterations in CXCL12 and their receptor. Indeed, they concluded that alterations of CXCL12 and their receptor and at less extend changes in CX3CL1–CX3CR1 expression will probably be normalized following chronic treatment with anti-depressants [79].

Moreover, several clinical studies found correlations between elevated levels of circulating inflammatory chemokines and depressive symptoms in patients with major depressive disorder [73, 80–83]. According to the results of a comprehensive meta-analysis, peripheral concentrations of a number of chemokines including CCL2, CCL3, CCL4, CCL11, CXCL4, CXCL7, and CXCL8 can potentially discriminate between individuals with depression and those without [84]. Additionally, Ślusarczyk et al. (2016) provided a comprehensive account of the role of chemokines in processes underlying depressive disorder [85].

### Challenges of cytokine research in psychiatry

There are some difficulties faced by researchers in conducting cytokine research in psychiatry. The major problem seems to be heterogeneity of the obtained results. In other words, research outcomes are conflicting and challenging to interpret [24]. Moreover, research in this area is largely based on measurement of cytokine levels in the periphery and it is not completely clear how serum or plasma levels of cytokines reflect the situation in the brain [47]. Compellingly, results of studies that examined both peripheral and CFS levels of IL-6 found no correlations between the mentioned measures; thus, suggesting that peripheral levels of IL-6 may not directly reflect central IL-6 levels [86, 87].

It is also noteworthy that some environmental, social, biological, and medical factors may influence peripheral cytokine changes. For instance, one of the characteristics of obesity is chronic inflammation with the increased circulating levels of cytokines [88, 89]. Indeed, adipose tissue is reported to build up and activate lymphocytes and macrophages that secrete inflammatory factors [89, 90]. Interestingly, obese people show behavioral symptoms such as MDD and cognitive dysfunction at an increased rate in comparison with the general population [91, 92]. Therefore, one may argue that alterations in cytokine levels are somehow unspecific [24]. Additionally, there is a considerable overlap in cytokine values between patients in the acute phase of depression, patients in remission, and patients who are recovered [24]. Although the use of cytokines as potential biomarkers of depression has been discussed frequently in various studies, cytokine changes have been reported in other psychiatric disorders as well. For instance, increased levels of pro-inflammatory cytokines have been reported in generalized anxiety disorder [93–95], obsessive-compulsive disorder [96], posttraumatic stress disorder [97, 98], and sleep disorder [99].

Moreover, cytokine levels change during pharmacotherapy of depression. Indeed, it has been suggested that treatment with anti-depressants can potentially lead to alteration in peripheral levels of cytokines. According to the results of a meta-analysis, anti-depressants, overall, cause decrease in peripheral levels of IL-6, IL-10, and TNF-α [100]. However, anti-psychotics which are used in psychotic depression, especially those with the highest risk of weight gain (e.g., clozapine and olanzapine), cause significant increase in the blood levels of pro-inflammatory cytokine [101]. Additionally, mood stabilizers such as lithium and carbamazepine have also been linked with an increase in the peripheral levels of cytokines [102]. In sum, as cytokine signaling often exhibits pleiotropic, redundant, synergistic, and antagonistic effects, it seems to be advisable to consider all cytokines that work together or against each other and therefore, take into account the whole range of cytokines instead of a single one.

### On the role of interleukin-6 in depression

#### IL-6 alterations in depression

A growing body of evidence suggests that IL-6 has a crucial role in pathogenesis of depression [3] and is the most consistently increased cytokine in blood samples of MDD patients [38, 103]. The first promising evidence for the role of IL-6 in occurrence of depression is most probably provided by a longitudinal study in which children with higher circulating levels of IL-6 at age 9 were found to be at a 10% greater risk of developing MDD by age 18, compared to the general population or children with low IL-6 levels. Indeed, the researchers concluded that inflammation and high IL-6 levels possibly predate the occurrence of depression [104]. Another evidence for potential role of IL-6 in depression is that peripheral levels of IL-6 were found to be positively correlated with symptom severity in anti-depressant non-responders [105].

Stress-based preclinical models of depression showed that IL-6 levels are increased following the onset of depression-associated behaviors. Rodents who were exposed to chronic mild stress exhibited anhedonia and elevated circulating levels of pro-inflammatory cytokines including IL-6 [106, 107]. Moreover, in another study on male Wistar rats, serum levels of pro-inflammatory cytokines including IL-6 were reported to be higher in acute and restraint stress compared to non-stressed rats [108]. It is also noteworthy that some studies reported no significant alteration in peripheral levels of IL-6 in chronic mild stress models of depression [109]. Nevertheless, they reported elevated CNS levels of other inflammatory markers which probably reflects a time-dependent shift from peripheral to central cytokine activation or potential transport of the peripheral cytokines into CNS [109]. Another promising evidence was provided by studies on IL-6 knockout mice. Indeed, they were reported to be resistant to the development of depression-like phenotype following long-term light deprivation in the constant darkness, proposing a functional role for IL-6 in stress susceptibility [110]. Moreover, Ślusarczyk et al. (2015) found evidence for the role of prenatal stress as a priming factor that could exhibit effects on microglial cells and consequently lead to depressive-like disturbances in adult rat offspring. According to their results, the release of pro-inflammatory cytokines including IL-6 is enhanced in microglia obtained from prenatally stressed animals compared to control animals [78].

In fact, not every individual exposed to prolonged or acute stress develops a psychiatric disorder [111]. According to previous research, vulnerability to repeated social defeat stress is predicted by differences in IL-6 levels in the innate peripheral immune system [112]. Following induction of social defeat stress, two thirds of mice were reported to show depression-like behavior measured by social avoidance, anhedonia, circadian system disruptions, and metabolic changes [113] together with elevated activation of pro-inflammatory cytokines such as IL-6 [112]. Indeed, higher degrees of elevation in peripheral IL-6 levels of susceptible mice were reported in comparison with resilient mice. Moreover, it was found that this increase occurs within 20 min of the first social defeat. Interestingly, mice that later became susceptible had higher number of leukocytes and those leukocytes produced more levels of IL-6 following stimulation via LPS ex vivo [112]. Additionally, studies with non-social stress-based models found evidence for the functional role of IL-6 in the development of stress susceptibility. In these models, animals were exposed to a controllable or uncontrollable stress (e.g., shock), and their ability to actively escape a subsequent stressor was measured. According to the results, 20% of animals who were exposed to uncontrollable stress were found susceptible and developed learned helplessness and the rest were found to be resilient. Interestingly, susceptible animals showed elevated levels of peripheral IL-6 together with anhedonia in contrast to resilient animals [114].

Clinical studies have also revealed that patients with MDD have increased levels of plasma and serum concentrations of pro-inflammatory cytokines including IL-6 in comparison with healthy controls [24, 100, 115, 116]. It should be noted that three meta-analyses verified increased peripheral IL-6 levels in MDD patients compared to healthy volunteers [38, 116, 117]. Nevertheless, there are also studies reporting no significant differences in IL-6 levels in MDD patients compared to healthy volunteers [118]. However, one may argue that different subtypes of depression and certain depressive symptoms should be taken into account. For instance, Rudolf et al. (2014) compared IL-6 levels among patients with atypical and typical depression and healthy controls. According to their results, IL-6 levels were significantly increased in patients with atypical depression and not in typical MDD patients compared to healthy controls [119]. Additionally, Rush et al. (2016) studied peripheral levels of IL-6 and TGF-β in 55 melancholic depressed patients. They were found to have significantly higher baseline IL-6 levels compared to healthy controls. Moreover, these elevated levels of IL-6 did not normalize following electroconvulsive therapy (ECT) [120]. A recent systematic review conformed Rush et al.’s results. In the mentioned review, authors found that peripheral IL-6 levels are increased in patients with melancholic depression in comparison with controls [121]. Moreover, Maes et al. (1997) examined serum levels of IL-6 and IL-1 receptor antagonist in patients with chronic, treatment resistant depression both before and after subchronic treatment with anti-depressants. According to their results, subchronic treatment with anti-depressants had no significant impact on serum levels of IL-6; nevertheless, it decreased serum soluble IL-6R levels significantly [122].

### Effects of IL-6 on neurotransmitters’ synthesis, signaling, metabolism, and function

The effects of cytokines on neurotransmitters have been studied extensively [49, 123]. Cytokines and their signaling pathways (e.g., p38 mitogen activated protein kinase) are reported to exhibit significant impacts on metabolism of multiple neurotransmitters such as serotonin, dopamine, and glutamate; thus, influencing their synthesis, release, and reuptake [49]. Indeed, cytokines can decrease synthesis of serotonin via activating the enzyme indoleamine 2,3 dioxygenase (IDO) which breaks the precursor of serotonin (i.e., tryptophan) to kynurenine (KYN) instead of metabolizing tryptophan to serotonin; thus, leading to serotonin depletion [3]. The process of serotonin depletion has been long associated with major depression [124, 125]. Moreover, cytokines can modulate serotonin signaling via elevating the expression and function of monoamine transporters. These transporters are known to re-uptake serotonin [126, 127].

IL-6 is known to influence neurotransmission by modulating the behavioral output of the brain; however, the exact mechanism is unknown. A previous study showed that IL-6 directly controls the levels of serotonin transporter (SERT) and therefore influences serotonin reuptake. Indeed, the researchers concluded that IL6-induced modulation of serotonergic neurotransmission through the signal transducer and activator of transcription 3 (STAT3) signaling pathway contributes to the role of IL6 in depression [128]. The activity of SERT forms serotonergic transmission which is implicated in depressive behavioral changes and pathophysiology of the disease [129]. By intensifying dopaminergic and serotonergic turnover in hippocampus and frontal cortex, IL-6 influences neurotransmission of catecholamines [130]. It seems that noradrenaline is not affected by IL-6; however, noradrenaline itself can induce expression of IL-6 in glial cells [131]. IL-6 together with other pro-inflammatory cytokines can activate kinurenine pathway which is involved in glutamatergic neurotransmission [132].

### Effects of IL-6 levels on brain morphology in depression

Previous studies showed that elevated levels of pro-inflammatory cytokines such as IL-6 may affect neurogenesis [133] and neural plasticity [134]. Imaging studies have shown that specific brain regions such as basal ganglia (which is involved in motor activity and motivation), the dorsal anterior cingulate cortex (ACC) (which has a central role in generation of anxiety), and the subgenual ACC (which is known to be involved in the development of depression) are influenced by cytokines [135, 136]. Additionally, high IL-6 expression levels demonstrated neuropathologic manifestations including neurodegeneration [137, 138].

There are many studies in which positron emission tomography (PET) has been applied to test translocator protein (TSPO) binding, a marker of neuroinflammation, in order to study neuroinflammatory hypothesis of depression [139–143]. According to their results, neuroinflammation was present in various regions of the brain (e.g., neocortical grey matter, frontal cortex, prefrontal cortex, anterior cingulate cortex, insula, temporal cortex) as well as the hippocampus [139–143].

In a recent study, Kakeda et al. (2018) evaluated possible relationship between serum levels of IL-1β, IL-6, IFN-γ, and TNFα and brain morphology in terms of brain cortical thinning and hippocampal subfield volumes during the first depressive episode in drug-naïve patients with MDD using a whole-brain SBM analysis. They found a significant inverse correlation between prefrontal cortex (PFC) thickness and serum IL-6 level in MDD patients. Indeed, high serum levels of IL-6 were correlated with reduced left subiculum and right CA1, CA3, CA4, GC-DG, subiculum, and whole hippocampus volumes in MDD patients. Additionally, thickness of the superior frontal and medial orbitofrontal cortices in patients with depression was significantly decreased compared to healthy controls. Since PFC contains high concentrations of IL-6 receptors, IL-6 mediated neurotoxicity might happen under conditions in which high serum IL-6 levels are present (i.e., early stages of MDD). Consequently, the authors advocated that the neuroinflammatory status in the early stage of MDD is associated with changes in the brain gray matter and IL-6 probably plays a key role in the morphological changes observed in the PFC during early stages of the disease. It is also noteworthy that serum IL-6 was the only cytokine among the tested cytokines that showed significant differences between the patients and controls in their study. Indeed, serum IL-6 levels were found to be significantly higher in MDD patients than in healthy controls [144]. In another study, Frodl et al. (2012) investigated possible effects of changes in the glucocorticoid and inflammatory systems on hippocampal volumes in patients with MDD. According to their results, MDD patients showed increased IL-6 levels and smaller hippocampal volumes compared to healthy controls. Positive effects of messenger RNA (mRNA) expression of glucocorticoid-inducible genes and further inverse effects of IL-6 concentration, on hippocampal volumes were also reported. Thus, they concluded that increased expression of IL-6 can probably predict decreased hippocampal volume [145].

**Fig. 1:**
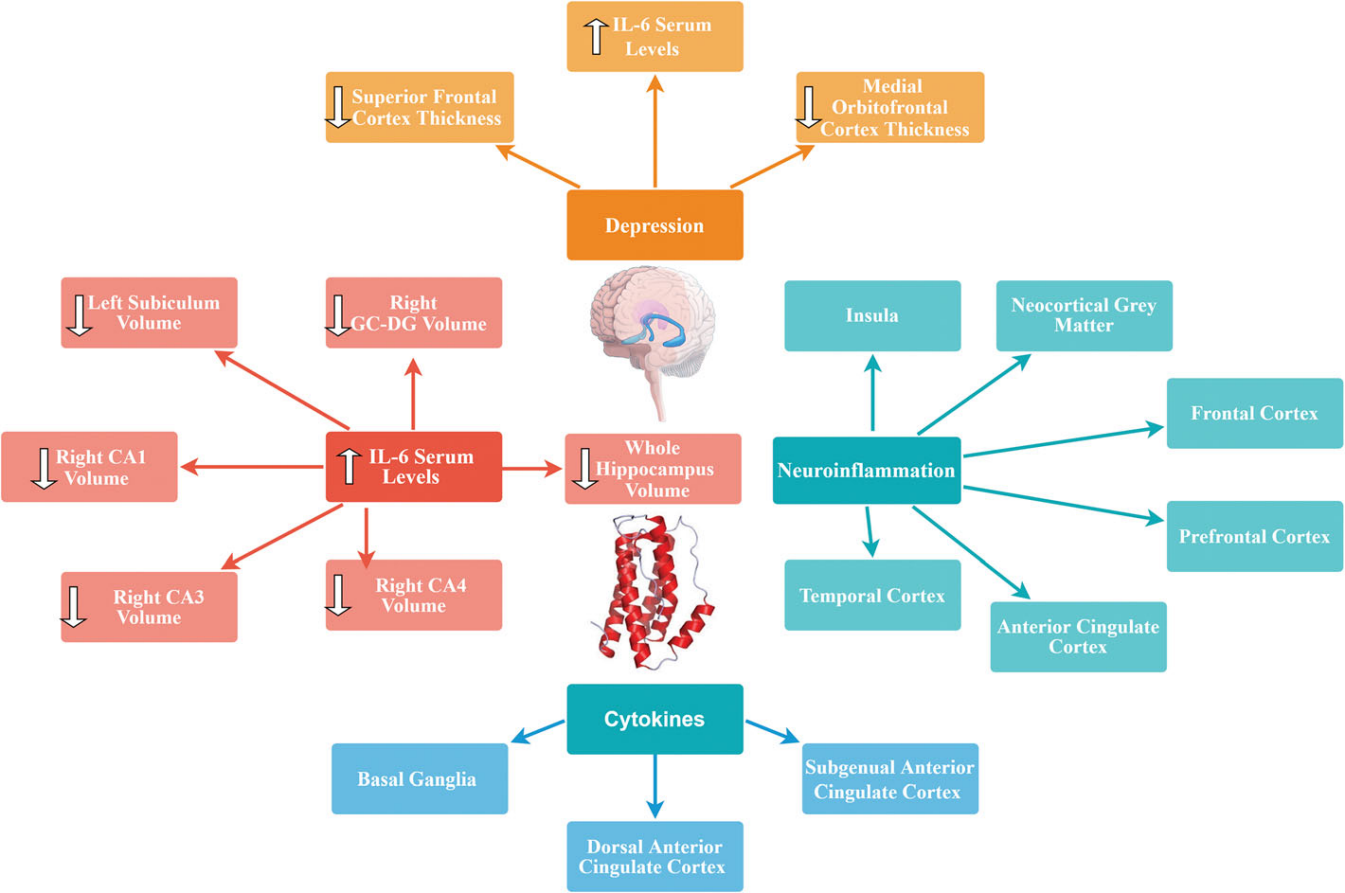
Effects of cytokines including IL-6 on brain morphology

As already mentioned, there is considerable amount of evidence regarding the central role of the highly plastic, stress-sensitive hippocampal region in pathogenesis of depression [146]. Indeed, grey-matter structures, including the hippocampus are vulnerable to atrophy in depression [147, 148]. Hippocampal volume reductions are most probably the result of remodeling of key cellular elements, involving retraction of dendrites, loss of glial cells, and decreased neurogenesis in the dentate gyros [149]. Factors underlying this cellular remodeling are known to be stress-induced increased levels of glucocorticoids, which are implicated in decreased neurogenesis [150]. Moreover, increased activity of the HPA axis resulting in decreased levels of glucocorticoids combined with resistance to glucocorticoid-induced negative feedback control is commonly observed in depression [151]. This dysregulation of glucocorticoid secretion along with the increased activity of excitatory neurotransmitters can potentially lead to cellular remodeling (which can be reversible) and hippocampal neurons cell death in patients with depression [152]. Since hippocampus has been identified to have a role in negative feedback inhibition of glucocorticoids, remodeling or neuronal damage may lead to less efficient inhibitory control of the corticotrophin-releasing hormone, resulting in elevated amounts of circulating glucocorticoids and further damage of the hippocampal neurons [153]. Taken together, it seems that further studies are required to elucidate the physiological mechanisms in which IL-6 might exert changes in the brain grey matter. A brief overview of the effects of cytokines including IL-6 on brain morphology is shown in Fig. 1.

### Blockade of IL-6 and its receptor in the periphery as a potential therapeutic option in MDD

Growing body of evidence suggests that abnormalities in the immune system are most probably relevant to pathogenesis and potential novel treatment of psychiatric disorders. Previous studies showed that alterations in the peripheral IL-6 levels might contribute to depressive-like behavior in animal studies [3, 112, 114, 154]. Moreover, IL-6 knockout mice showed resistance to development of depressive-like behavior [155] which gives further evidence for possible role of IL-6 pathogenesis of depression. High peripheral levels of IL-6 are even more apparent in patients with treatment-resistant depression. Additionally, correlations have been found between decrease in IL-6 levels and alleviation of depressive symptoms in patients who were responsive to the pharmacotherapy [156]. Moreover, results of a study on 222 stroke patients indicated significant associations between IL-6 periphery levels and development of MDD within 2 weeks and at 1 year following stroke. Furthermore, significant correlations were found between statin use and IL-6 on the presence of a depressive disorder at the 1st year. Indeed, preventive effects of treatment with statins (which are known to possess anti-inflammatory properties and potently reduce the cytokine-mediated IL-6 release [157]) against post-stroke depression was confirmed [158]. Thus, suppression of IL-6 activity could possibly lead to clinical recovery and may be considered as a novel pharmacotherapeutic option. Utilizing IL-6 receptor antibodies (for instance, Tocilizumab) or IL-6 antibodies (for instance, Sirukumab or Siltuximab) for reduction of IL-6 activity seems to be a novel strategy.

Blockade of IL-6 receptor by the humanized anti-IL-6 antibody, Tocilizumab has been used in treatment of rheumatoid arthritis (RA) [159–163] and systemic juvenile idiopathic arthritis [164–166]. Extensive clinical studies have established both short-term and long-term efficacy and safety of Tocilizumab [±conventional disease-modifying anti-rheumatic drugs (DMARDs)] in adults with moderate to severe RA. Additionally, Tocilizumab was shown to be effective as a monotherapy in patients with systemic juvenile idiopathic arthritis and also in patients whose disease has been refractory to other therapies [164]. Moreover, the safety profile of tocilizumab was reported to be consistent over time and also consistent with safety profile of other immunomodulatory agents [162]. It is also important to note that oral tocilizumab has been shown to inhibit experimental autoimmune encephalitis by elevating Th2 anti-inflammatory cytokines and decreasing pro-inflammatory Th1 cytokines [167]. With regard to crucial role of IL-6 in regulating metabolic homeostasis, side effects such as significant weight gain followed by hypertrygliceridemia and hypercholesterolemia may be observed in patients treated with tocilizumab [168]. Blockade of IL-6 trans-signaling, while classical IL-6R signaling stays intact seems to be crucial for the goal of maintaining gut mucosal integrity and epithelial regeneration [65]. Indeed, few randomized clinical trials were conducted on anti-depressant properties of tocilizumab. According to the results of a recent meta-analysis of anti-depressant activity of anti-cytokine therapies, treatment with tocilizumab showed statistically significant improvements in depressive symptoms [169].

Another promising human monoclonal antibody against Il-6, namely Sirukumab has been reported to be a safe and well-tolerated agent, capable of modulating the immune response in healthy populations as well as in patients with inflammatory disorders (e.g., rheumatoid arthritis). It targets the IL-6 signaling pathway by inhibition of both the pro- and anti-inflammatory effects of IL-6 [170]. Effects of Sirukumab on cytokine networks provide a well-founded rationale for its potential use in pharmacotherapy of psychiatric disorders promising possible advantages across varying domains of the biobehavioral research criteria [171]. In a phase 2, double-blind, placebo-controlled trial, the efficacy of Sirukumab and Siltuximab on depressive symptoms was studied in patients with rheumatoid arthritis or multicentric Castleman’s disease respectively. Compared with placebo, both IL-6 neutralizing antibodies were found to make significantly greater improvements on depressive symptoms in the patients [172]. Results of a recent mega-analysis of 18 randomized, placebo-controlled clinical trials of efficacy of immunomodulatory drugs on depressive symptoms in patients with inflammatory disorders demonstrated promising results (*N* = 10,743 participants). According to their findings, anti-IL-6 antibodies (sirukumab and siltuximab) had large and statistically significant effect sizes on core depressive symptoms before correction for physical health outcomes. Additionally, their effects remained significant in non-responders for the primary disease states evaluated [173]. Although further research is needed in this area, potential application of anti-IL-6 antibodies could possibly open new avenues in pharmacotherapy of MDD.

### Possible role of IL-6 together with gut microbiota in pathogenesis of depression

The human intestine harbors nearly 100 trillion bacteria [174] consisting assemblages of microorganisms associated with various niches in and on the body with long-term implications to health [175]. Evidence is emerging regarding correlations of microbial activities with progressive structural and functional processes in the brain of both animal models and humans [175]. There is a large body of evidence for the role of gut microbiota composition in pathogenesis of depression [176–180]. Moreover, there is growing body of literature for the influence of the gut microbiome on cytokine signaling [181, 182].

The dominant gut microbial phyla are known to be *Firmicutes* and *Bacteroideteds* [183, 184]. The *Firmicutes*/*Bacteroideteds* ratio is of great relevance in signaling human gut microbiota status [185]. For instance, increased levels of *Firmicutes*/*Bacteroideteds* ratio have been reported in patients suffering from irritable bowel syndrome (IBD) and seem to have some correlations with development of depression and anxiety [186, 187]. Additionally, *Firmicutes*/*Bacteroideteds* ratio is associated with overall alterations in bacterial profiles at different life stages [185]. In a novel study, researchers reported decreased *Firmicutes*/*Bacteroideteds* ratio in mice following social defeat stress; thus, proposing possible role of *Firmicutes*/*Bacteroideteds* in depressive-like behavior. Furthermore, administration of anti-mouse IL-6 receptor antibody (MR16-1) attenuated the decreased ratio of *Firmicutes*/*Bacteroideteds* in susceptible mice. Thus, the researchers concluded that anti-mouse IL-6 receptor antibody may have anti-depressant-like effects by normalizing the *Firmicutes*/*Bacteroideteds* ratio via modulation of the immune system [3].

Decreased number of *Oscillospira* was detected in patients with depression [180] which suggests for possible role of *Oscillospira* in pathogenesis of depression. Two animal studies yielded same results. A recent study investigated therapeutic effects of finasteride on depressive-like behavior in rats together with 1 month of treatment withdrawal. Withdrawal from finasteride was associated with increased depressive-like behavioral responses. Therapeutic use of finasteride was linked with elevations in the phylum Bacteroidetes and in the family Prevotellaceae, and withdrawal was found to be correlated with decreases in the family Ruminococcaceae and the genera *Oscillospira* and *Lachnospira* [188]. In another study, socially stressed mice developing depression-like symptoms showed increases at the genus level of fecal *Oscillospira*. Interestingly, IV administration of anti-mouse IL-6 receptor antibody (MR16-1) normalized depression-like behavior and resulted in significant decrease in *Oscillospira* levels towards pre-stressor levels [3]. Moreover, increased number of *Sutterella* was reported in fecal samples [189] and intestinal biopsy samples of children with Autism spectrum disorder [190]. Additionally, elevated number of *Staphylococcus* and *Sutterella* were found in mice following social defeat stress. It is likely that *Staphylococcus* and *Sutterella* play a role in the depressive-like behavior via infection-induced inflammation. Interestingly, administration of anti-mouse IL-6 receptor antibody resulted in attenuation of elevated number of *Staphylococcus* and *Sutterella* following social defeat stress in mice [3].

**Fig. 2:**
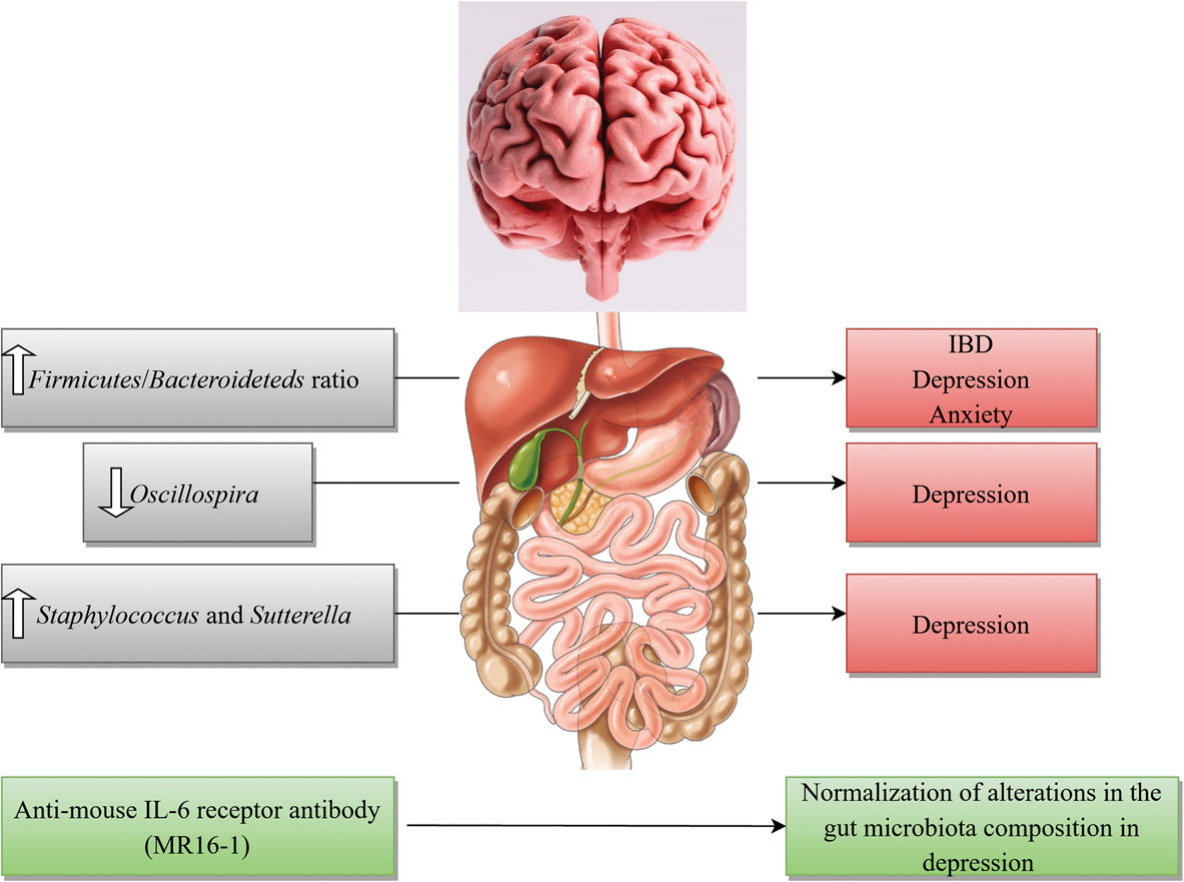
A brief overview of the role of IL-6 together with gut microbiota in pathogenesis of depression

These findings advocate that peripheral IL-6 may have a significant role in pathogenesis of MDD and blockade of IL-6 receptor in the periphery may exhibit rapid-onset effects by attenuating the altered composition of gut microbiota. Taking into account the role of gut-microbiota in immunomodulation, it is highly probable that gut-microbiota-brain- axis plays a role in anti-depressant actions of treatment with anti-IL-6 receptor [3]. A brief overview of the role of IL-6 together with gut microbiota in pathogenesis of depression is shown in Fig. 2.

### Elevated levels of IL-6 in patients with COVID-19 infection

The world-wide effect of the coronavirus disease 2019 (COVID-19) pandemic is enormous and is not solely limited to the increased mortality and morbidity rates, but also extends into the mental health of the global population [191]. Considerable amount of clinical data is emerging regarding the manifestation of depression in patients during [192–194] and post-COVID 19 infection [192–195]. It is estimated that about 48% of confirmed COVID-19 cases displayed overt psychological symptoms such as depression and often expressed feelings of regret, loneliness, helplessness, and irritation [196].

There is growing body of literature regarding dual role of IL-6 in COVID-19 infection and depression [192]. Normal plasma levels of IL-6 in adults range between 1 and 10 pg/ml; whereas in a systemic inflammation this amount increases to several ng/ml [197] and even higher concentrations were reported in COVID-19 patients [198]. Indeed, cytokine release syndrome (CRS) is common in COVID-19 patients and increased levels of serum IL-6 have been identified to be significantly associated with acute respiratory distress syndrome (ARDS), respiratory failure, and poor disease outcome in numerous studies [192, 199–201]. Studies suggest that new onset depression is most probably caused by inflammation initiated during the active phase of the infection leading to a cytokine surge [202]. Indeed, severe acute respiratory syndrome coronavirus 2 (SARS-CoV-2) infects primarily human monocytes and dendritic cells causing dendritic cell dysfunction, leading to T cell apoptosis and exhaustion; thus, contributing to the immunopathology [203]. Alpert et al. (2020) described two cases of COVID-19 patients with elevated amounts of IL-6 (25 pg/mL and 26.7 pg/mL respectively) who were diagnosed with major depressive disorder (according to the Diagnostic and Statistical Manual of Mental Disorder, 5th Edition (DSM-5)) during COVID 19 infection. Both patients’ depressive symptoms subsided about 6 weeks after initiation of anti-depressant pharmacotherapy and normalization of the inflammatory cytokines [192]. The authors concluded that lower cytokine activity ameliorates depressive symptoms as normalization of IL-6 plasma levels decreased depression with or without anti-depressants [192]. Moreover, Benedett et al. (2020) studied effects of treatment with cytokine-blocking agents on the psychopathological status of the patients with COVID-19 infection. Their results were in favor of the protective effects of treatment with cytokine-blocking agents in early phases of COVID-19 against the later onset of depression [204].

It is indeed crucial to maintain a multidisciplinary approach in management of the psychological effects of this debilitating pandemic. Treatment strategies addressing the immunopathology of SARS-CoV-2 infection will be promising during the acute phase of the disease [192, 205]. Currently, there are few studies considering psychological and neuropsychiatric implications of COVID-19; however, it is very likely to expect an increased incidence of mental pathologies both during and post-COVID-19 infection.

## Conclusion

Preclinical and clinical studies present strong evidence that inflammation is altered in a subset of patients with MDD and there is mounting body of literature for the role of pro-inflammatory cytokines namely IL-6 in pathophysiology of depression. Nevertheless, there still exists gap in our understanding of the mechanisms by which IL-6 signaling and its molecular components could possibly contribute to depression manifestation. A number of humanized monoclonal antibodies are undergoing clinical trials for potential pharmacotherapy of mood disorders. Biologics including IL-6 receptor antibodies or IL-6 antibodies are currently approved to treat inflammatory disorders such as RA and are undergoing clinical trials as a novel target for MDD treatment. However, these novel therapeutic targets may also raise the possibility of potential side effects. By investigating the interface of peripheral cytokines, namely IL-6 and brain cellular processes contributing to depression, one might be able to develop novel therapeutic options for treatment of mood disorders by sequestering and preventing this peripherally derived inflammatory marker from acting upon mood circuits in the CNS. In sum, therapeutic deficiency in treatment outcomes reflects the growing demand for revitalizing psychiatric therapeutics with novel options that could potentially open new avenues in treatment of this debilitating disorder and enhancement of patients’ quality of life.

## Data Availability

Not applicable.

## Abbreviations

ACC: Anterior cingulate cortex
ARDS: Acute respiratory distress syndrome
BBB: Blood-brain barrier
CNS: Central nervous system
COVID-19: Coronavirus disease 2019
CRS: Cytokine release syndrome
CTRA: Conserved transcriptional response to adversity
DMARDs: Disease-modifying anti-rheumatic drugs
DSM-5: Diagnostic and Statistical Manual of Mental Disorder, 5th Edition
ECT: Electroconvulsive therapy
Gp130: Glycoprotein 130
GPCRs: G-protein-coupled receptors
HPA: Hypothalamic-pituitary-adrenal
IDO: Indoleamine 2,3 dioxygenase
IFN: Interferon
IL: Interleukin
IL-6R: IL-6 receptor
JAK/STAT: Janus kinase/signal transducer and activator of transcription
KYN: Kynurenine
MDD: Major depressive disorder
MAPK: Mitogen-activated protein kinase
mRNA: Messenger RNA
NF-κB: Nuclear factor kappa-light-chain-enhancer of activated B cells
PFC: Prefrontal cortex
RA: Rheumatoid arthritis
SARS-CoV-2: severe acute respiratory syndrome coronavirus 2
SERT: Serotonin transporter
STAT3: Signal transducer and activator of transcription 3
sIL-6R: Soluble IL-6R
TGF: Transforming growth factor
T_H_2: T helper type 2
T_H_1: T helper type 1
Th: T helper cell
TNF: Tumor necrosis factor
TSPO: Translocator protein
Treg: Regulatory T cells
